# Selected Essays on Children's Diseases

**Published:** 1842-07

**Authors:** 


### Art. XIX.

1. *Analekten über Kinderkrankheiten.* Band III. und IV.—*Stuttgart*, 1837.

*Selected Essays on Children's Diseases.* Vols. III. and IV.—*Stuttgard*, 1837.

2. *Von Mezler, Sammlung auserlesener Abhandlungen über Kinderkrankheiten.* IX. Hefte—*Prag.* 1836-40.

*Von Mezler's Collection of the most important Essays on the Diseases of Children.* Nine Parts.—*Prague*, 1836-40. 8vo.

The first two volumes of the Stuttgard Selection on Children's Diseases were noticed with well-merited commendation in this Review for October, 1836. Since that time not only has the work been brought to a termination in a manner highly creditable to its editor, but a second series of essays, arranged on a similar plan, has been published at Prague under the superintendence of Dr. v. Mezler.

The nature of these compilations was sufficiently explained in the notice of the first two volumes of the Analekten. Without endeavouring, therefore, to enumerate all the subjects treated of in the volumes now before us, we shall seek to cull for our readers whatever may appear to be most novel or important.

*Dropsy.* The first paper on which we light is on a peculiar form of dropsy, (Analekten, Heft ix. p. 42,) by Dr. Wolff, of Bonn, a physician deservedly eminent in the Rhine provinces for his skill in the management of infantile diseases. This affection is not very unusual, Dr. Wolff having met with above a hundred cases in his own practice during six years. He has never observed it after the age of puberty, but the children attacked by it were in most instances between their second and fifth year. It is usually ushered in by general indisposition, with loss of appetite, and an irregular state of the bowels. The tongue is slightly furred, and the patient suffers from occasional pain in the abdomen, with some degree of fever and acceleration of pulse. These symptoms are frequently so slight as to be overlooked by the parents, but at other times attention is excited by the severity of the abdominal pain and the frequent occurrence of vomiting. After the lapse of from five to fourteen days, during which it often happens that drastic purgatives are given to the child, under the supposition that it is suffering from worms, the pains become constant, the febrile symptoms more strongly marked, the loss of appetite is complete, and the abdomen grows tumid, and on a careful examination yields a distinct sense of fluctuation. Among the symptoms of the disease, Dr. Wolff attaches considerable importance to a peculiar tumidity about the root of the nose, and the value of this sign is confirmed by Professor Nasse.

This form of abdominal dropsy does not reach so great a degree as is often attained by ascites in the adult, and it is never associated with œdema of the extremities. Hence its real nature may be overlooked. If, however, the disease be left to itself, the extremities of the child grow emaciated by degrees, till at length the skin hangs around them in folds; fluctuation becomes gradually more obscure without any diminution occurring in the size of the abdomen, the patient's strength fails, universal emaciation takes place, the bowels are now purged, now constipated, irregular accessions of fever come on, and the little sufferer pines away into its grave.

This fatal event, however, is unusual, for the disease appears very accessible to the influence of proper treatment, and hence Dr. Wolff has never had the opportunity of making a post-mortem examination. He conceives that the affection consists in a chronic form of peritonitis, and accordingly employs in its earlier stages leeches to the abdomen, together with small doses of calomel. In some instances digitalis was combined with the calomel, and in the more advanced stages of the disease great benefit appeared to result from a combination of small doses of digitalis and cream of tartar. Whenever the disease has reached its second stage it becomes especially important to make a correct diagnosis, lest the practitioner should be betrayed into the employment of stimulating remedies, which invariably produce an injurious effect.

Dr. Wolff expresses his conviction that many of the cases of atrophia infantum, in which no scrofulous disease exists, are produced by the injudicious management of this form of chronic peritonitis.

*Stomacace.* Passing over two essays on mesenteric disease, the one by Dr. Wendt of Breslaw, the other by Guersent of Paris, and extracted from the Dictionnaire de Médecine, we come to a paper on stomacace, by Jörg of Leipsic, and to another on gangræna infantilis, by Richter. The latter paper is judiciously compiled by the editor from various memoirs by Richter, but its subject is more familiar to the medical reader than that peculiar affection to which Jörg gives the name of stomacace. It occurs in children between the periods of dentition and puberty, and consists in the eruption of several small vesicles containing a transparent fluid, and surrounded by an inflamed area, on the tongue, the gums,and inner surface of the lips. In the course of a few days these vesicles become converted into small excavated ulcers, which are not coated by lymph, and furnish only a very scanty secretion of pus. The whole lining membrane of the mouth is at the same time more or less inflamed, the secretion from the salivary glands is increased in quantity as well as altered in character, giving out a very offensive smell, and the ulcers are so painful that all movements of the mouth, as in mastication or speaking, are avoided by the children as much as possible. Loss of appetite, slight fever, and a constipated state of the bowels, are associated with the local affection; but under judicious management, the disease usually runs its course in from eight to twelve days. Purgative medicines and appropriate diet, and washing the mouth frequently with aromatic gargles are the means requisite for its cure. Professor Jörg states that he has never known a case in which this affection terminated unfavorably, and we refer to it now partly from having seen it occasion considerable alarm to persons who did not sufficiently understand its differences from the far more formidable disease, *cancrum oris*.

*Malignant scarlatina.* In the eleventh number, besides many other very interesting papers, we meet with the account, by Professor von Ammon of Dresden, of an epidemic of malignant scarlet fever which prevailed in that city during six months, at the close of 1831 and beginning of 1832. In October 1831, several cases of *scarlatina simplex* appeared in one of the suburbs of Dresden, and ran a favorable course, but in other parts of the city many children died from a cerebral affection which was unattended with any trace of an eruption, but proved very rapidly fatal. In the middle of December cases of scarlet fever became much more numerous, and in January 1832, the disease was prevailing epidemically in all parts of the city. It attacked both sexes, and persons of all ages, and medicine did not seem to exert much influence on its course, death or recovery frequently occurring when least anticipated.

The onset of the disease was sometimes announced by the usual premonitory symptoms; but often persons were attacked by pneumonia, and expectorated blood, till with the appearance of the rash of scarlet fever all signs of disease of the respiratory organs disappeared. Children were sometimes seized while at play with violent headach; they fell into a sleep succeeded by coma or convulsions, and died within twenty-four hours. In others the eruption suddenly broke out while they were apparently in perfect health, and the fever ran its course without the supervention of a single bad symptom. The exanthema presented very various appearances, and its eruption was frequently accompanied with an extremely dangerous affection of the nares, throat, and respiratory organs, from which an ichorous secretion was poured out. The heat of the skin was great and pungent, the pulse was extremely frequent, and, though large, extraordinarily feeble. In those who died during the fever, head affections were the most frequent cause of the fatal result, but sequelæ of all kinds were very numerous and severe, and the dropsy which followed desquamation was especially to be dreaded.

Great turgescence of the vessels of the brain and congestion of the dependent parts of the lungs were generally found after death. In the body of one patient the whole substance of the heart showed marks of inflammation, which had terminated in gangrene. Several bullæ existed on the surface of the liver, and there were large ecchymoses on various parts of the intestines. The whole of the corpse gave out an insupportably gangrenous odour. These appearances were met with only in one case; but in all, the right cavities of the heart had a peculiar red colour, and the heart as well as the large arterial trunks contained large, firm, fibrinous coagula.

The treatment of the epidemic presents nothing of particular moment. Depletion was never found to be useful; but in many of the worst cases great benefit resulted from the administration of large doses of the carbonate of ammonia, as six or eight grains every hour.

Dropsy occurred in a great proportion of the cases. It was frequently accompanied with violent palpitation and other signs of affection of the heart, requiring the free use of local depletion. Antiphlogistic measures and mild diuretics led to the recovery of the patient in many instances; but not unfrequently, after the dropsy had been got rid of, and many days or even weeks of tolerable health had elapsed, inflammation was again set up in some internal organ, and purulent effusions were found after death in the pleura or pericardium or between the muscles.

If the inflammatory affection of the heart was overlooked at the outset, it often happened that the dropsy disappeared; but organic disease of the heart remained behind, under which the patients sank after some severe and protracted suffering.

The twelfth number occupies the whole of the fourth volume, which contains nearly five hundred pages. It opens with two essays on the Diagnosis of Infantile Diseases, by Valleix and Professor Naumann. Next follow Dr. Gregory's article on Smallpox, from the Cyclopædia of Practical Medicine, and Professor Wendt's essay on Scarlet Fever, somewhat abridged from his Manual of Children's Diseases. There are besides many other papers in this volume of which we would gladly present our readers with an abstract, but we must leave space for some notice of V. Mezler's judicious collection of essays.

Dr. v. Mezler has followed a plan similar to that adopted by the editor of the Stuttgard *Analekten*, but has wisely avoided, except in a very few cases, extracting the same papers. General observations on the diagnosis and therapeutics of children's diseases, by various writers, with an account of Goelis's practice at the Vienna Dispensary for Children, occupy the first number. The second number contains some general remarks on the diseases of children and their remedies, and two essays, one by Formey, on the encephalitis of children, the other by Hinze, on the greater frequency now than formerly of cerebral affections in early life.

*Hydrocephalus.* This is not the place for an examination into the correctness of Formey's theoretical views; of the value of his practical observations there can be no doubt. The points to which he attaches most importance, as aiding diagnosis in the earliest stages of acute hydrocephalus, are an eruption of a strophuloid character on the outer side of the upper arm, sometimes on the cheeks and lips; the appearance of peculiar, glistening, micaceous particles in the urine, of which Coindet speaks; the changeableness of the child's temper and peculiarity of its cry; staggering in walking, and frequent falling; inclination to sickness and actual vomiting, drowsiness without sleep; and the little impression produced by medicine, especially by purgatives. Such are the symptoms by which the disease is characterized during that stage when we may hope to cure it. The occurrence of effusion and the consequent great increase of danger are marked by the restlessness of the child giving place to a state of apathy interrupted by occasional cries, but from which it cannot be roused except by raising its head from the pillow. The eye now loses its sensibility to light, the pupil becomes preternaturally dilated, and the children lie in a condition resembling sleep with their eyelids half open. The vomiting occurs much more seldom, or ceases altogether, and the children devour in a hasty manner anything that is put into their hand. The pulse becomes slow and irregular, the bowels are confined, and the fæces solid and dark.

These symptoms are all of great value, though one or two of them we think are not of such universal application as they would appear to be from the above statement. The dilatation of the pupil is, we are convinced, the sign least of all to be relied on; for it not only varies as to the time of its occurrence, but it is not even observed in all cases. The slowness of the pulse, too, is sometimes wanting; its occasional irregularity we conceive to be of considerably greater importance, though we do not regard it as a symptom peculiar to the stage of effusion.

Formey is a great advocate of cold affusion on the head, which he directs to be repeated every hour or every two hours; and states that its continuance during several days may be requisite. The evidence of such men as Formey and Heim in favour of this practice would carry great weight, even though its good effects were not attested by many subsequent writers. The practitioner, however, should well count the cost before he begins to employ this remedy, and should above all make sure that the parents will second him in carrying out a plan which has the appearance of being needlessly cruel. With regard to the best time for its employment, we refer our readers to some very sensible observations by Dr. Münchmeyer, in the Hannoversche Annalen, of which an abstract was given in the October Number of this Journal. (Vol. XII. 559.)

The sixth number contains a paper by Professor Nasse, of Bonn, on the same subject as that by Hinze, and like everything written by that distinguished pupil of the famous Reil, it bears the impress of much research and thought. It does not, however, admit of condensation.

The eighth number contains several essays on measles and scarlet fever, from which we select one by the late Dr. Heim, of Berlin, on the differential diagnosis of scarlet fever, rubeolæ, (the Rötheln of German writers,) and measles.

*Rubeolæ*, (*Rötheln.*) Dr. Paterson, of Edinburgh, recently drew the attention of the profession in Great Britain to this variety of the exanthemata, in a paper published in the Edinburgh Journal for October, 1840. The subject, however, is of importance sufficient to claim a notice here, especially as no one has detailed the characteristics of rubeolæ with such minute accuracy as Heim. Heim is disposed to regard the disease as a variety of scarlatina: Dr. Wagner of Schlieben, however, states, in a paper originally published in Hecker's Annalen, that neither measles nor scarlet fever exercise any preservative influence in protecting a person from

*rötheln*. Of the two diseases, scarlatina certainly bears a much closer resemblance to rötheln than is presented by measles. Both scarlet fever and rubeolæ are characterized by great rapidity of pulse and by febrile symptoms, which often run very high. In both, the eruption usually appears within twenty-four hours after the invasion of the premonitory symptoms, and in both, sore throat is of frequent occurrence. Scarlatina, however, is sometimes unattended by sore throat, and at other times the affection of the throat is unaccompanied with any eruption; but in *rötheln* neither the angina nor the exanthema are ever absent. The character of the eruption of rötheln affords the chief means of discriminating between these diseases. Like the eruption of scarlet fever, it usually appears simultaneously upon the whole body; and, like it, is not accompanied with any sensible elevation of the skin, such as distinguishes the eruption of measles. But its colour is somewhat darker than that of the eruption of scarlet fever, and it is not subject to that sudden disappearance which occasionally occurs in the course of that disease. The characters which it puts on are twofold; consisting either of innumerable little dots, one line or a line and a half in diameter, of a somewhat irregular form, and presenting a well-defined outline; or the patches are destitute of a distinct boundary, and, if the disease is severe, coalesce so as to bear some resemblance to the rash of scarlet fever. The rash of rubeolæ, like that of scarlatina, disappears under pressure, but on the pressure being remitted the distinct spots of rubeolæ become evident, and from them the general redness extends. No such spots, however, appear if the finger is removed from the skin of a scarlet fever patient, but the redness returns without any such central point being observed.

The subject of rubeolæ requires still further investigation than has ever yet been bestowed upon it. There are many important differences between the statements of Heim and those of Dr. Paterson with reference to the diagnosis of the disease. Authors, too, are by no means agreed as to whether *rötheln* is a variety of measles or of scarlet fever, or whether it is, as many suppose, a really independent disease.

Our stock of essays is not nearly exhausted, but our space is; and we have room only most heartily to commend both the Stuttgard and Prague *Analekten* to all our readers, as forming by far the most valuable collection of essays on children's diseases with which we are acquainted.

---

